# Supplementary material for: Trends in the conduct and reporting of clinical prediction model development and validation: a systematic review
Source: J Am Med Inform Assoc. 2022 Jan 19;29(5):983–9. doi: 10.1093/jamia/ocac002 (PMC9006694; doi:10.1093/jamia/ocac002)
Supplement: ocac002_supplementary_data [file ocac002_supplementary_data.zip › Appendix B.docx]

# APPENDIX B. STUDY SELECTION FLOW DIAGRAM


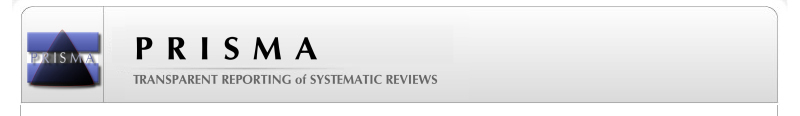
**PRISMA 2009 Flow Diagram**

Duplicates removed
(n = 3,697)

Records identified through database searching
(n = 9,932)

- Embase: 4,915
- Medline: 3,421
- Web-of-Science: 1,346
- Cochrane Library: 169
- Google Scholar: 81

Studies included for data extraction
(n = 422)

Full-text articles assessed for eligibility
(n = 1,075)

Full-text articles excluded
(n = 653)

- Not using EHR data (n = 159)
- Methodological focus (n = 140)
- Evaluating predictor associations (n = 95)
- Not prognostic prediction (n = 86)
- Not patient-level prediction of a particular clinical outcome (n = 77)
- Other article types (n = 41)
- Model updating study (n = 27)
- External validation study (n = 22)
- Full text unavailable (n = 6)

Records screened
(n = 6,235)

- Embase: 4,870
- Medline: 1,002
- Web-of-Science: 184
- Cochrane Library: 102
- Google Scholar: 77

Records excluded
(n = 5,160)

## Identification

## Eligibility

## Included

## Screening
